# Attenuating Effect of *Chlorella* Extract on NLRP3 Inflammasome Activation by Mitochondrial Reactive Oxygen Species

**DOI:** 10.3389/fnut.2021.763492

**Published:** 2021-10-08

**Authors:** Yuya Nakashima, Kazuhito Gotoh, Soichi Mizuguchi, Daiki Setoyama, Yurie Takata, Toshihiro Kanno, Dongchon Kang

**Affiliations:** ^1^Department of Clinical Chemistry and Laboratory Medicine, Graduate School of Medical Sciences, Kyushu University, Fukuoka, Japan; ^2^Department of Research and Development, Chlorella Industry Co., Ltd., Fukuoka, Japan

**Keywords:** pyrin domain-containing protein 3 (NLRP3) inflammasome, *Parachlorella beijerinckii*, mitochondrial reactive oxygen species (mtROS), IL-1b, macrophages

## Abstract

The NOD-like receptor family, pyrin domain-containing protein 3 (NLRP3) inflammasome has been linked to the pathogenesis of a wide variety of human diseases. Although many drugs and inhibitors have been developed to treat NLRP3-associated diseases, only limited clinical data support their efficacy and safety. *Chlorella*, a unicellular green alga that is widely and safely used as a food supplement, contains various antioxidants. In this study, we obtained a fat-soluble extract from *Chlorella* (CE) and demonstrated that it reduced NLRP3 inflammasome activation by inhibiting mitochondrial reactive oxygen species and caspase-1 activation. In addition, CE supplementation attenuated lipopolysaccharide-induced interleukin 1β transcription through activation of hypoxia-inducible factor 1α *in vitro* and *in vivo*. As *Chlorella* is a safe and useful food supplement, it may be a practical pharmacological approach for treating NLRP3-driven diseases.

## Introduction

The NOD-like receptor (NLR) family protein NOD-, LRR-, and pyrin domain-containing protein 3 (NLRP3) is an intracellular sensor molecule that detects many pathogen and host-derived factors (1, 2). Anomalous NLRP3 inflammasome activation is linked to the development of many diseases, including cryopyrin-associated periodic syndromes, sepsis, gout, osteoarthritis, Alzheimer's disease, diabetes, atherosclerosis, steatohepatitis, and colitis (2–5). However, there are currently no effective, safe, and selective therapeutic approaches for these diseases that enable inhibition of the NLRP3 inflammasome. Therefore, drugs or supplements that safely inhibit the NLRP3 inflammasome are needed.

Activation of the NLRP3 inflammasome depends on two functionally distinct steps: priming and activation (2). The priming step (signal 1) begins with the recognition by pattern recognition receptors (PRRs) of extracellular molecules such as lipopolysaccharide (LPS: TLR4 agonist). LPS stimulation increases nuclear factor-κB (NF-κB)-mediated NLRP3 and pro-interleukin (IL)-1β expression. Enhanced glycolysis and hypoxia inducible factor (HIF)-1α activation by LPS stimulation supports increased Il-1b transcription (6, 7). Upon activation, NLRP3 recruits and binds to apoptosis-associated speck-like protein containing a CARD (ASC). NLRP3 and ASC interact with the cysteine protease caspase-1 to form a super-complex termed the inflammasome (8, 9). Inflammasome activation triggers the self-cleavage and activation of caspase-1, converting pro-IL-1β and pro-IL-18 to their mature forms. In particular, mitochondrial dysfunction and the release of mitochondrial reactive oxygen species (mtROS) are additional key upstream events related to NLRP3 activation (10, 11). Therefore, decreasing mtROS leads to suppression of NLRP3 inflammasome activation.

*Chlorella* is a unicellular green alga with biological and pharmacological properties that are important for human health (12). It contains a variety of nutritional components, being enriched in proteins, fatty acids, dietary fibers, chlorophylls, minerals, vitamins, and carotenoids. Several studies have shown that *Chlorella* supplementation ameliorates hyperlipidemia, diabetes, atherosclerosis, muscle atrophy, dementia, and cancer (12–15). Because *Chlorella* supplement contains various components, it is unclear which component(s) is effective at improving these disorders.

Previous studies have shown that consumption of carotenoid-rich foods and supplements reduces the incidence or risk of various diseases (16, 17). Additionally, the consumption of carotenoids has been reported to have protective effects against several diseases such as atherosclerosis, diabetes, dementia, and age-related macular degeneration (18–21). *Chlorella* contains high concentrations of carotenoids (e.g., lutein, zeaxanthin, and β-carotene) and the carotenoids in *Chlorella* may be therapeutic for these diseases. Previous studies showed how to extract carotenoids from *Chlorella* (22, 23). However, it is unclear how carotenoid-rich extract from *Chlorella* (CE) affect *in vitro* and *in vivo*. We hypothesized that CE contain strong antioxidants and have an anti-inflammatory effect. Therefore, we purify CE and examine the anti-inflammatory effect of CE *in vitro* and *in vivo*.

In this paper, we describe that CE attenuated LPS-induced HIF-1α activation and Il-1b transcription. CE also suppressed NLRP3-dependent caspase-1 activation and IL-1β secretion by regulating mtROS.

## Materials and Methods

### Preparation of Carotenoid-Rich Extract From *Chlorella*

*Chlorella* (*Parachlorella beijerinckii*) dried powder (Chlorella Industry Co. Ltd.) was extracted in methanol and chloroform (1:2) at room temperature overnight in the dark. The extracts were filtered through filter paper. The filtrated extracts were evaporated under reduced pressure and dissolved ethanol (EtOH). For saponification, 50% KOH (w/v) was added to the extract solutions for 2 h. After saponification, 3% NaCl (w/v), distilled water, and diethyl ether were mixed and extracted carotenoids were transferred to the upper layer. The residue was repeatedly extracted with diethyl ether several times. The upper layers were evaporated and dissolved in tetrahydrofuran (THF) to prepare a stock solution. The stock solution was stored in the dark at −80°C. Carotenoids in CE were measured by high performance liquid chromatography (HPLC) (LC-20AT, Shimadzu Corporation). HPLC analysis was performed by Japan Food Research Laboratories. HPLC measurement conditions were as follows: Detector, UV-VIS Detector SPD-20AV (Shimadzu Corporation); for lutein and zeaxanthin; Column, Luna silica (4.6 × 250 mm, 3 μm, Phenomenex, Inc.); Mobile phase, hexane-acetone (82:18, v/v); Flow rate, 1.2 mL/min; Wavelength, 450 nm; for α-carotene and β-carotene; Column, Inertsil ODS-4 (4.6 × 250 mm, 5 μm, GL sciences Inc.); Mobile phase, acetonitrile-methanol-THF-acetic acid (55:40:5:0.1, v/v/v/v); Flow rate, 1.5 mL/min; Wavelength, 455 nm. The carotenoid composition of CE is shown in Supplementary Table S1.

### Reagents

LPS and antibodies against β-actin were purchased from Sigma-Aldrich (USA), and lutein, β-carotene, and α-tocopherol were obtained from Wako Pure Chemicals (Japan). Zeaxanthin was purchased from Cayman Chemical Co. The reagents used in this study are shown in Supplementary Table S2.

Antibodies against p38 (#9212), phospho-p38 (#4631), JNK (#9258), phospho-JNK (#4668), Erk1/2 (#9102), phospho-Erk1/2 (#4370), IκB (#4814), phospho-IκB (#2859), NF-κB p65 (#8242), phospho-NF-κB p65 (#3033), HIF-1α (#36169), Caspase-1 (#24232), Cleaved Caspase-1 (#89332), NLRP3 (#15101), ASC (#67824), and AIM2 (#63660) were purchased from Cell Signaling Technology, USA. The antibodies used in this study are shown in Supplementary Table S3.

### Cell Cultures and *in vitro* Stimulation

The mouse macrophage cell line RAW264 was obtained from the Riken BioResource Center (Tsukuba, Japan). To isolate peritoneal macrophages (pMACs), we injected mice intraperitoneally with 4 mL of 4% (vol/vol) thioglycollate solution. Peritoneal exudate cells were isolated from the peritoneal cavity 3.5 days after intraperitoneally injection. Collected cells were incubated for 2 h in 100 mm cell culture dishes and washed five times with phosphate-buffered saline (PBS). We used the adherent cells as pMACs for the experiments.

Bone marrow-derived macrophages (BMDMs) were prepared as described elsewhere (24). Bone marrow cells were collected from femurs and tibias. Then, bone marrow cells were cultured for 6–8 days with M-CSF (10 ng/mL; PeproTech, USA) in complete medium, DMEM (Sigma-Aldrich, USA) supplemented with 10% FBS, penicillin (Nacalai Tesque, Japan), and streptomycin (Nacalai Tesque, Japan).

6 × 10^5^ Macrophages (RAW264 cells, pMACs, and BMDMs) were seeded in 6-well plate and treated with Mock or CE (0.3 or 1.5 μM) for 16 h in complete DMEM medium with M-CSF. Then, these cells were stimulated with LPS (10 ng/mL or 100 ng/mL) without washing. For inflammasome activation, macrophages were primed with LPS (100 ng/mL) before stimulation with ATP or nigericin (NLRP3 inflammasome activators) as described (11). An enzyme-linked immunosorbent assay (ELISA) of mouse IL-1β was performed using a Mouse IL-1β ELISA kit (BioLegend, USA) in accordance with the manufacturer's instructions.

### Treatment With CE and Carotenoids *in vitro* and *in vivo*

CE contained multiple carotenoids, and therefore the extract was adjusted based on the concentration of lutein the most abundant in CE; Supplementary Table S1. As a stock solution was used in all experiments, the concentration of lutein in CE was diluted to a final concentration of 1 mM (dissolved in EtOH:DMSO:THF; 1:1:6). Additionally, each pure carotenoid and vitamin E (lutein, zeaxanthin, β-carotene, and α-tocopherol) were dissolved and diluted to the same concentration in the solvent. In *in vitro* experiments, the stock solutions of CE, carotenoids, and vitamin E were added to medium with 0.15% EtOH:DMSO:THF (1:1:6). The same amount of solvent was dissolved in the untreated control (Mock). For *in vivo* experiments, the same CE stock solution was added to PBS with 0.15% EtOH:DMSO:THF (1:1:6) and mice were injected intraperitoneally.

### Real-Time PCR

After stimulation or treatment with LPS, macrophages, mouse spleen, and liver were collected and washed in PBS, then resuspended in RLT buffer. Total RNA was extracted from macrophages, mouse spleen, and liver using an RNeasy Mini Kit (Qiagen), in accordance with the manufacturer's instructions. After treatment with RNase-free DNase I (QIAGEN, Germany), RNA samples were reverse-transcribed using PrimeScript™ RT Reagent Kit (TAKARA, Japan), in accordance with the manufacturer's instructions. Quantitative real-time RT-PCR analysis was performed using specific primers (Supplementary Table S4). The expression of the genes was determined by qPCR with a thermal cycler (StepOne Plus; Applied Biosystems). PCR cycling conditions were one cycle of 95°C for 1 min, 40 cycles of 95°C for 15 s and 60°C for 1 min, one cycle at 72°C for 1 min.

### Immunoblotting Analysis

For direct immunoblotting, pMACs and BMDMs were lysed with cell lysis buffer (Cell Signaling Technology) as described (25). The lysate was eluted with sample buffer and boiled for 5 min at 97°C. SDS-PAGE was performed with 8–15% polyacrylamide gels. The gels were transferred to Immobilon-P membranes (Merck Millipore). The membranes were blocked and incubated with the appropriate primary antibody overnight at 4°C in Can Get Signal Solution 1 (Toyobo Co., Ltd.) (Supplementary Table S3). Signals were visualized by chemiluminescence using a Clarity ECL Substrate (BIO-RAD) and an ImageQuant LAS4000 Mini image analyzer (GE Healthcare).

### Immunofluorescence Microscopy

After stimulation with LPS (10 ng/mL) for the indicated time periods, BMDMs were fixed with 4% paraformaldehyde/PBS for 15 min and permeabilized with 0.2% TritonX-100/PBS for 15 min as described (26). The cells were incubated with primary antibodies in 1% BSA/PBS for 1 h. Then, the cells were washed with PBS and incubated with an Alexa 488-labeled anti-rabbit secondary antibody for 1 h. The cells were washed and mounted on glass slides using Superfrost (Matsunami, Japan). Images were obtained under a fluorescence microscope (BZ-9000, KEYENCE, Japan).

### Metabolism Assays

BMDMs were analyzed using an XF-24 Extracellular Flux Analyzer (Seahorse Bioscience) as described (27). Briefly, BMDMs were seeded in XF-24 well culture plates (400,000 cells/well). At the specified time points, BMDMs were washed and analyzed in XF running buffer (unbuffered RPMI medium with 10 mM glucose, 10% fetal calf serum, and 2 mM L-glutamine) in accordance with the manufacturer's instructions to obtain real-time measurements of the ECAR. Analyses of the ECAR were performed in response to 10 ng/mL LPS.

### Transmission Electron Microscopy

BMDMs were fixed in 2.5% glutaraldehyde in 0.1 M cacodylate buffer at room temperature for 2 h as described (28). Samples were post-fixed in 0.1 M sucrose buffer with 1% OsO4 at 4°C for 2 h. Samples were dehydrated in a graded ethanol series. Ultrathin sections were prepared with an ultramicrotome (EM UC7, Leica) and stained with 2% uranyl acetate and lead citrate. The sections were imaged under a transmission electron microscope (Tecnai 20, FEI Co.).

### FACS Analysis

For mtROS measurement using MitoSOX as described (25), macrophages were incubated with ATP or nigericin and treated MitoSOX (5 μM, Thermo Fisher Scientific, #M36008) last 10 min, in accordance with the manufacturer's instructions. The supernatant was removed and resuspended in HBSS. Cells were analyzed by FACS. Data were acquired with a FACS Verse (BD Biosciences) and were analyzed with FACSuite software (BD Biosciences).

### Animal Experiments

Male C57BL/6J mice were purchased from Clea Japan. All mice were kept under specific pathogen-free conditions in the animal facility at Kyushu University. The animal protocols were approved by the Committee of Ethics on Animal Experiments, Faculty of Medical Sciences, Kyushu University.

Male mice (8–12 weeks old) were divided into four groups: (i) Mock; (ii) CE; (iii) Mock + LPS; (iv) CE + LPS. Mice were intraperitoneally injected with Mock (PBS) or CE (300 μg/kg body weight). At 16 h following Mock or CE injection, mice were intraperitoneally injected with PBS or LPS (2–5 mg/kg body weight) as described (26). Sixteen hours after LPS injection, the mice were anesthetized and blood, spleen, and liver samples were collected.

To examine mtROS *in vivo*, we injected mice intraperitoneally with 4 mL of 4% (vol/vol) thioglycollate solution. Mice were intraperitoneally injected with Mock (PBS) or CE (300 μg/kg body weight) 2.5 days after intraperitoneally injection of thioglycollate solution. Then, mice were anesthetized and intraperitoneally injected with LPS (5 mg/kg body weight) 3.5 days after intraperitoneally injection of thioglycollate solution. Four hours after LPS injection, pMACs were collected and cultured for 1 h and then washed five times with PBS. Then, pMACs were incubated with ATP or nigericin for 1 h and treated MitoSOX (5 μM) last 10 min. The supernatant was removed and resuspended in HBSS. Cells were analyzed by FACS. Data were acquired with a FACS Verse (BD Biosciences) and were analyzed with FACSuite software (BD Biosciences).

### Quantification and Statistical Analysis

Statistical analyses were performed using Microsoft Excel and GraphPad Prism. All experiments were performed in at least two independent biological replicates. The results are expressed as the average ± SD or ± SEM of the independent experiments. *P*-values were calculated using two-tailed Student's *t*-test.

## Results

### Supplementation of *Chlorella* Extract Inhibits Inflammatory Gene Transcription in Macrophages

**Figure 1:**
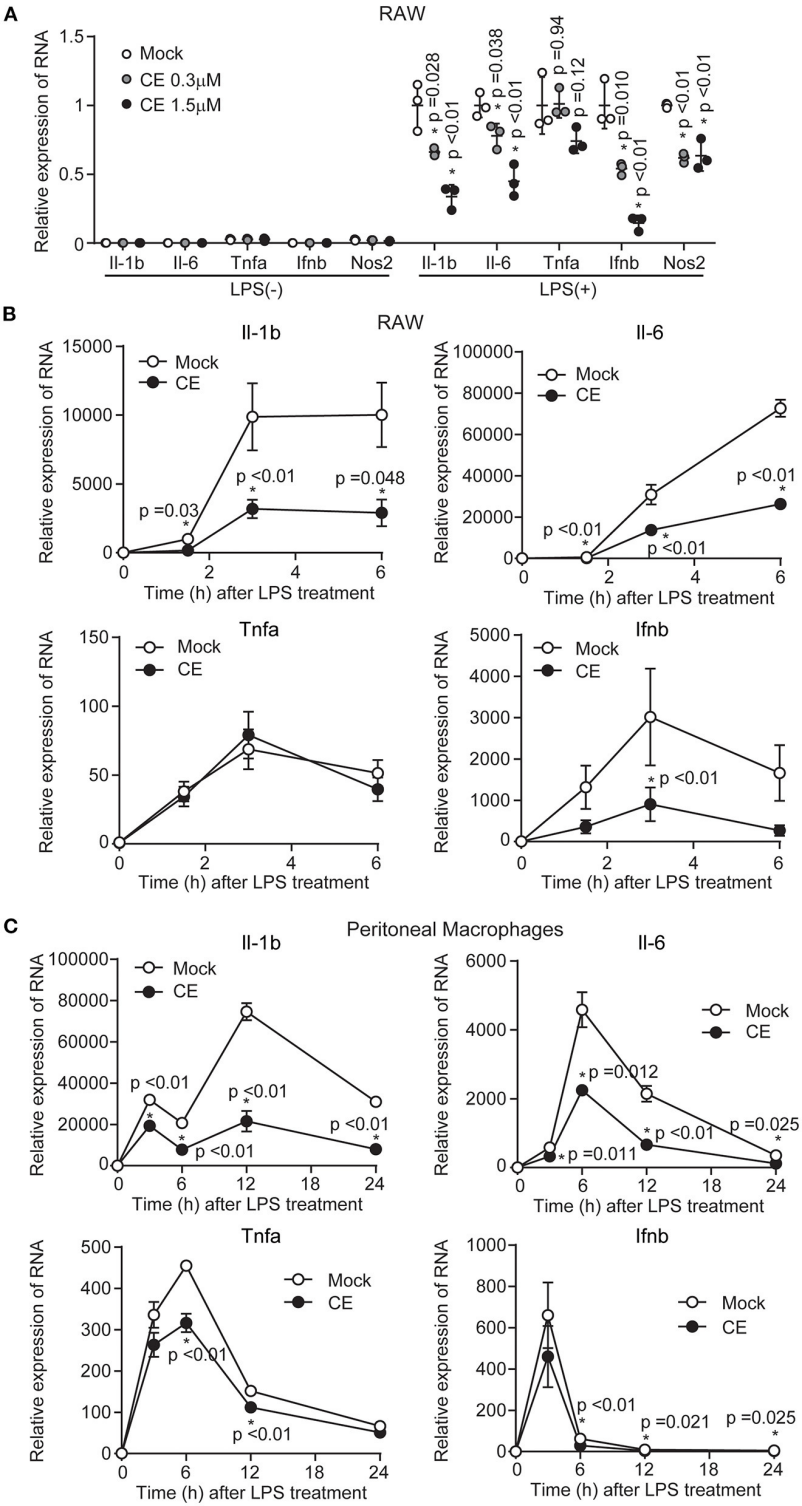
The effect of *Chlorella* extract on the expression of inflammatory genes in LPS-stimulated murine macrophages. **(A)** RAW264 cells were treated with Mock or CE for 16 h before being stimulated with LPS (100 ng/mL) for 3 h. The mRNA levels of Il-1b, Il-6, Tnfa, Ifnb1, and Nos2 of expressions were analyzed. **(B,C)** RAW264 cells and pMACs were treated with Mock or CE for 16 h before being stimulated with LPS (100 ng/mL) for the indicated time periods. The mRNA levels of Il-1b, Il-6, Tnfa and Ifnb1 of expressions were analyzed. All data were normalized expression of the gene encoding 18S ribosomal RNA (18S rRNA). Error bar show mean ± SEM. *p < 0.05 vs. Mock-treated groups. Data are representative of three independent experiments.

To confirm the anti-inflammatory effects of CE, we first examined the expression of cytokine genes induced by LPS in RAW264 and pMACs. Because our previous human study showed that plasma lutein concentration increased around 1.26 ± 0.49 μM after the daily oral intake of 9 g of dried powder from *Chlorella* (29), we treated macrophage cells with CE at a lutein-equivalent maximum amount of 1.5 μM (lutein: 1.5 μM, zeaxanthin: 0.123 μM, α-carotene: 0.019 μM, β-carotene: 0.091 μM, α-tocopherol: 0.163 μM) in medium for 16 h before LPS. CE significantly and dose-dependently inhibited the LPS induction of the expression of several inflammatory cytokine genes including Il-1b and Il-6 in RAW264 macrophages (*p* < 0.05; Figure 1A). In addition, the gene expression of Ifnb1 and Nos2 significantly decreased in RAW264 macrophages with CE after LPS stimulation (*p* < 0.05; Figure 1A). When we next compared the kinetics of cytokine gene expression induced by LPS, the expression of Il-1b and Il-6 was significantly downregulated in response to LPS in RAW264 macrophages (*p* < 0.05; Figure 1B) and pMACs (*p* < 0.05; Figure 1C) with CE. Although it is not clear whether CE has an inhibitory effect on LPS-induced gene expression of Tnfa and Ifnb1 in mouse macrophages (Figures 1B,C), we thought that CE specifically suppresses LPS-induced Il-1b transcription.

### Supplementation of *Chlorella* Extract Inhibits IL-1β Production in Macrophages

**Figure 2:**
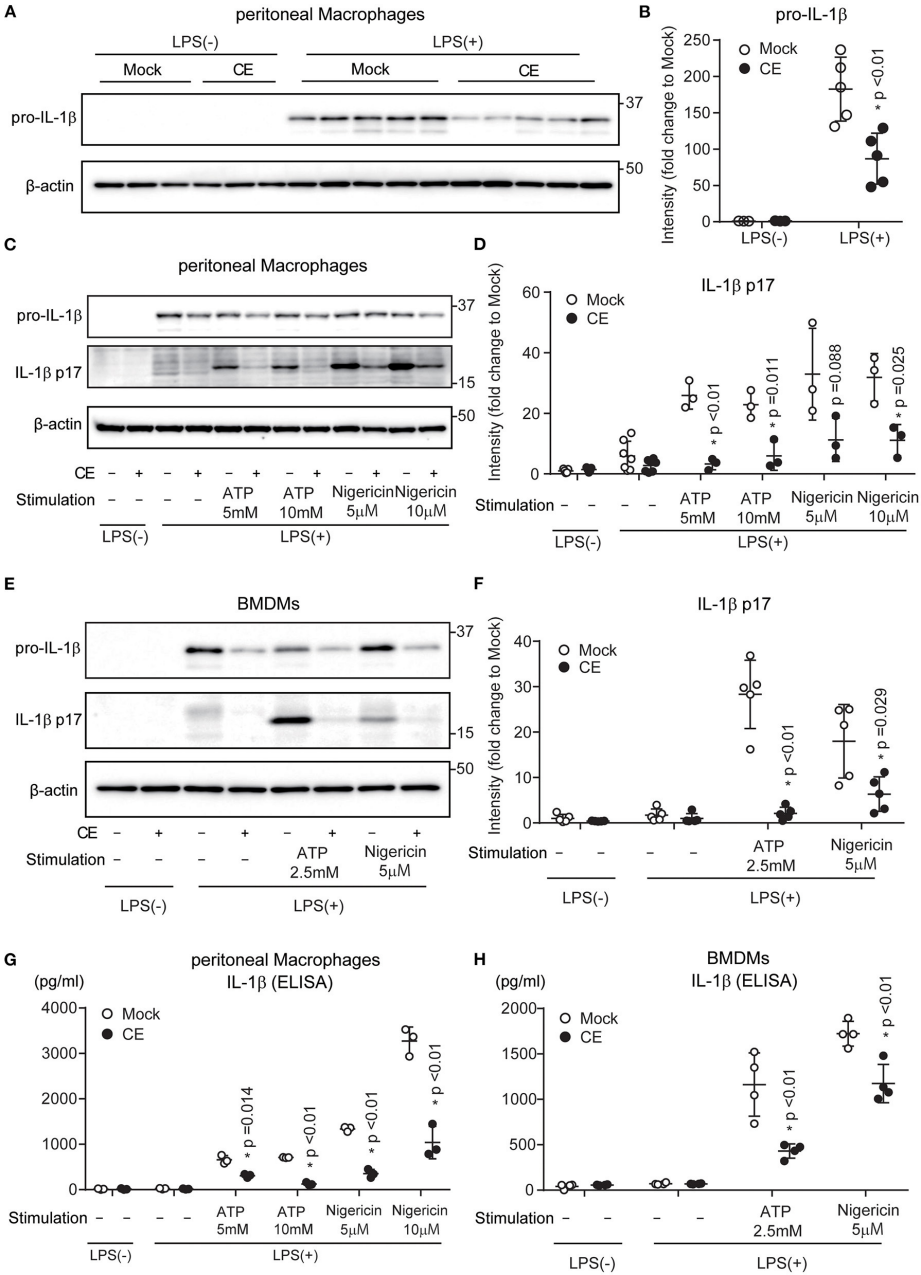
*Chlorella* extract inhibits IL-1β secretion in murine macrophages. **(A,B)** pMACs were treated with Mock or CE for 16 h before being stimulated with 100 ng/mL LPS for 4 h. **(C–H)** pMACs and BMDMs were treated with Mock or CE for 16 h prior to the addition of 100 **(C,D,G)** or 10 **(E,F,H)** ng/mL LPS for 4 h before stimulation with ATP or nigericin for 60 **(C,D,G,H)** or 30 **(E,F)** min. The protein levels of pro-IL-1β and IL-1β p17 were analyzed by western blotting [western blot image **(A,C,E)**, quantification **(B,D,F)**]. The levels of IL-1β in cell culture supernatants were analyzed by ELISA **(G,H)**. A representative western blotting quantification of pro-IL-1β **(B)** and IL-1β p17 **(D,F)** normalized to β-actin expression and relative to Mock only treated group. Error bar show mean ± SEM. *p < 0.05 vs. Mock-treated groups. Data are representative of at least three independent experiments.

Activation of the TLR4 signaling pathway increases the transcription of the Il-1b gene encoding pro-IL-1β and intracellular levels of the pro-cytokine. Pro-IL-1β is cleaved by caspase-1 and released as mature IL-1β from macrophages (30, 31). The processing and secretion of mature IL-1β via caspase-1 follow activation of the NLRP3 inflammasome. Consistent with the expression levels of mRNA (Figure 1C), CE supplementation reduced the amount of pro-IL-1β by LPS priming in pMACs (*p* < 0.01; Figures 2A,B). We next evaluated the effect of CE on IL-1β production in murine macrophages following NLRP3 inflammasome activation using ATP and nigericin. Western blotting showed that mature IL-1β was reduced in pMACs and BMDMs with CE following LPS/ATP (*p* < 0.05) and LPS/nigericin (Figures 2C–F). We also found that CE supplementation significantly inhibited ATP and nigericin-induced IL-1β secretion in mouse macrophages (*p* < 0.05; Figures 2G,H). Taking these findings together, CE supplementation inhibits IL-1β transcription and production in macrophages.

### *Chlorella* Extract Supplementation Attenuates LPS-Induced Il-1b Transcription Through HIF-1α Activation and Transnucleation

**Figure 3:**
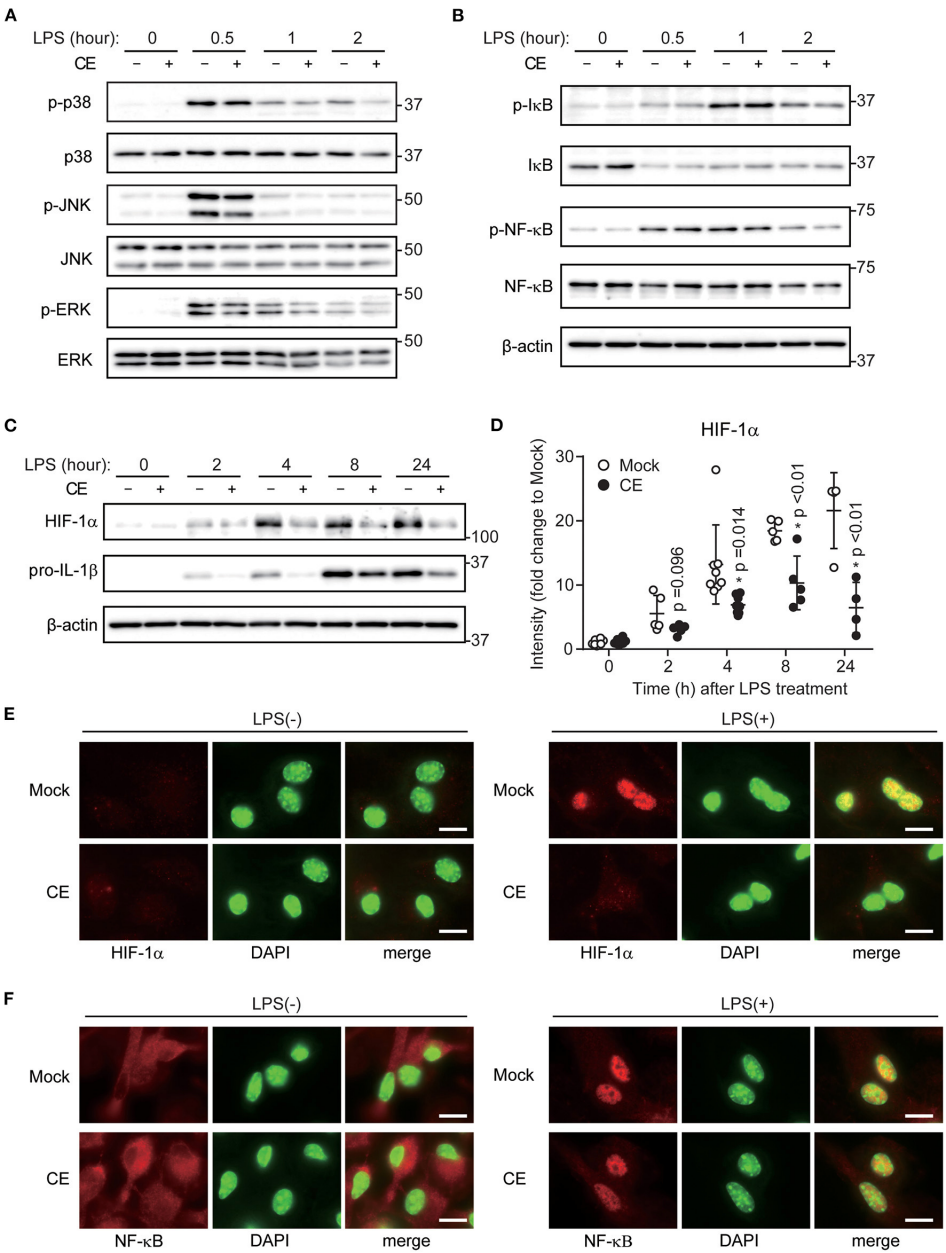
*Chlorella* extract inhibits LPS-induced HIF-1α activation. **(A–D)** BMDMs were treated with Mock or CE for 16 h before being stimulated with 10 ng/mL LPS for the indicated time periods. Whole cells lysates were analyzed by western blotting for total and phosphorylation of p38, JNK, ERK **(A)**, IκB, NF-κB **(B)**, HIF-1α and pro-IL-1β. Quantification of HIF-1α and pro-IL-1β **(D)** was normalized to β-actin expression and relative to Mock only treated group. **(E,F)** Subcellular localizations of HIF-1α **(E)** and NF-κB **(F)** were performed by immunohistochemistry. DAPI was used to stain nuclei. Scale bar, 10 μm. Error bar show mean ± SEM. *p < 0.05 vs. Mock-treated groups. Data are representative of at least three independent experiments.

LPS-induced activation of signaling cascades of the transcription factor NF-κB and mitogen-activated protein kinases (MAPKs) contributes to inflammatory gene activation. Supplementation of CE did not affect TLR4 downstream signaling, including activation of p38, c-Jun NH_2_-terminal kinase (JNK), extracellular signal-regulated kinase (ERK), NF-κB, and inhibitor of κB (IκB) in macrophages after LPS stimulation (Figures 3A,B; Supplementary Figures S1a,b). Several previous studies showed that LPS-induced IL-1b transcription was enhanced by increases of glycolysis and HIF-1α activation (6, 32). Therefore, we analyzed whether CE suppresses the enhancement of glycolysis and HIF-1α activation by LPS. Consistent with previous reports (33–35), LPS stimulation enhanced glycolysis without CE supplementation (Supplementary Figure S1c). CE supplementation did not affect LPS-induced glycolysis (Supplementary Figure S1c). In contrast, we found that CE suppressed LPS-induced HIF-1α activation in BMDMs 4 h after LPS stimulation (*p* < 0.05; Figures 3C,D; Supplementary Figure S1d). CAY10585 is a small-molecule inhibitor of HIF-1α activity (36). CAY10585 inhibited the LPS-induced increase in pro-IL-1β (Supplementary Figure S1d). In contrast, deferoxamine mesylate, an iron chelator and an activator of HIF-1, enhanced LPS-induced pro-IL-1β (Supplementary Figure S1d). Consistent with previous studies (6, 37), there was a positive correlation between the expression levels of HIF-1α and pro-IL-1β (Supplementary Figure S1d). Therefore, we thought that CE suppresses LPS-induced pro-IL-1β through HIF 1α activation. The gene promoter for IL-1β contains a HIF-1α-binding site. In addition, LPS-induced HIF-1α activation directly enhances IL-1β transcription (6). Thus, we next examined the nuclear translocation of HIF-1α and NF-κB by staining BMDMs with anti-HIF-1α and NF-κB antibodies together with DAPI staining of nuclei. Consistent with our findings (Figures 3C,D), LPS enhanced the expression level of HIF-1α in the absence of CE (Figure 3E). In addition, the majority of the increased HIF-1α was localized in the nucleus after LPS stimulation. In contrast, CE suppressed the activation and nuclear translocation of HIF-1α by LPS stimulation (*p* < 0.01; Figure 3E; Supplementary Figure S2a). Because CE did not affect the nuclear translocation of NF-κB by LPS stimulation (*p* > 0.05; Figure 3F; Supplementary Figure S2b), CE selectively inhibits the activation and nuclear translocation of HIF-1α by LPS stimulation.

### *Chlorella* Extract Supplementation Inhibits Mitochondrial ROS During NLRP3 Inflammation

**Figure 4:**
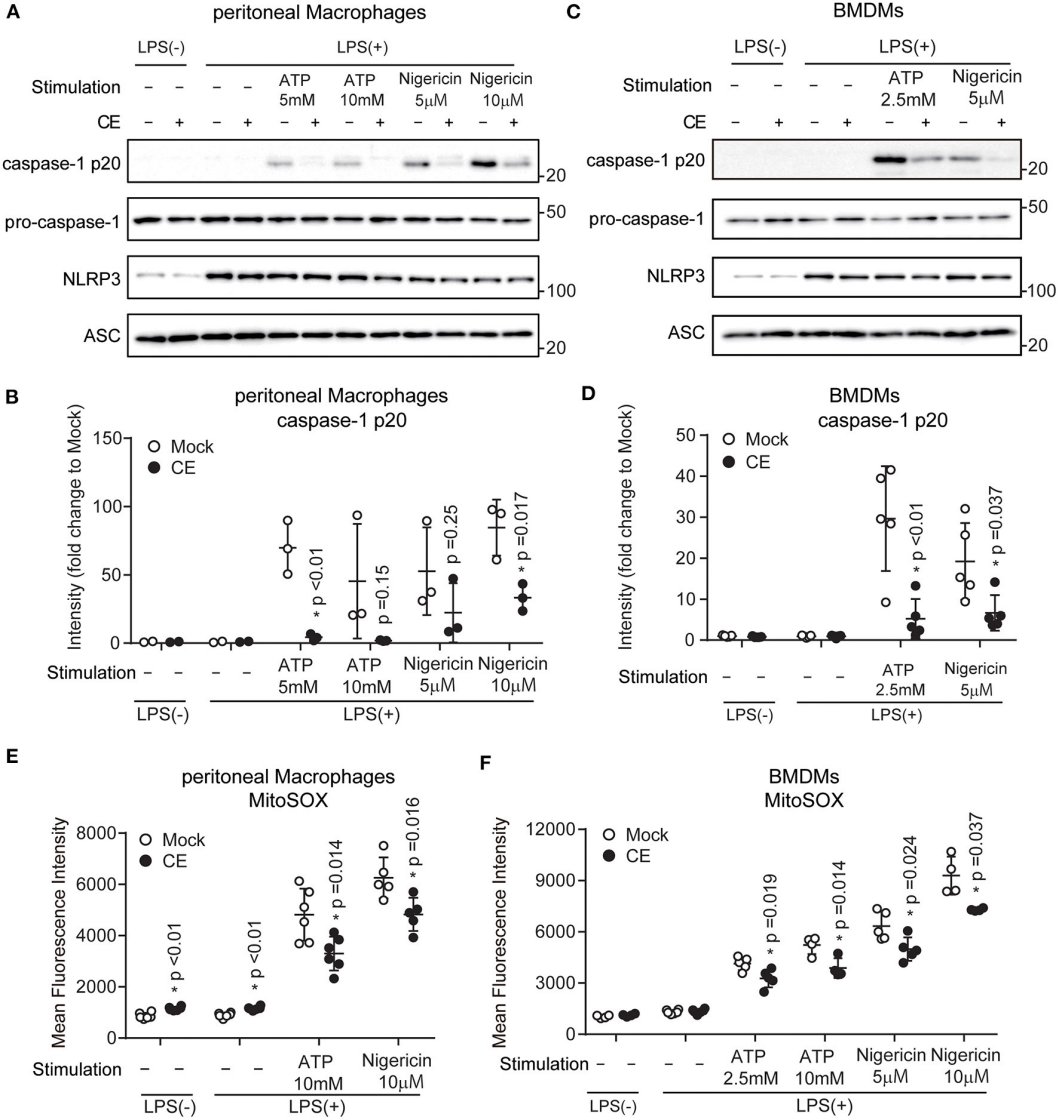
*Chlorella* extract inhibits mitochondrial ROS in NLRP3 inflammasome activation. **(A–F)** pMACs and BMDMs were treated with Mock or CE for 16 h prior to the addition of 100 **(A,B,E)** or 10 **(C,D,F)** ng/mL LPS for 4 h before stimulation with ATP or nigericin for 60 **(A,B,E)** or 30 **(C,D,F)** min. The protein levels of pro-caspase-1, caspase-1 p20, NLRP3 and ASC were analyzed by western blotting [western blot image **(A,C)**, quantification **(B,D)**]. A representative western blotting quantification of caspase-1 p20 **(B,D)** was normalized to β-actin expression and relative to Mock only treated group. Cells were stained with MitoSOX Red for 10 min and then were analyzed by FACS to measure mitochondrial ROS **(E,F)**. Error bar show mean ± SEM. *p < 0.05 vs. Mock-treated groups. Data are representative of at least three independent experiments.

The canonical inflammasomes are composed of at least three main components: an inflammatory caspase-1, an adapter molecule (such as ASC), and a sensor protein (such as NLRP1, NLRP3, NLRP12, NAIP1, NAIP2, NAIP5, or AIM2). The NLRP3 inflammasome is a protein complex formed by NLRP3, ASC, and pro-caspase-1 (38). Activation of the NLRP3 inflammasome leads to proteolytic activation of caspase-1, which triggers the cleavage and subsequent secretion of proinflammatory cytokine IL-1β (8). To confirm the inhibitory effects of CE on NLRP3 inflammasome activation, we examined whether CE inhibited the expression of NLRP3, ASC, and pro-caspase-1. Although CE did not reduce the levels of NLRP3, ASC, and pro-caspase-1 after LPS/ATP or LPS/nigericin activation, we found that supplementation of CE inhibited ATP (*p* < 0.05) and nigericin-induced caspase-1 activation (Figures 4A–D). MtROS are direct activators of the NLRP3 inflammasome by activating caspase-1 (39, 40). Because CE inhibits the NLRP3 inflammasome via caspase-1 activation, we assessed mtROS production using the mitochondrially targeted probe MitoSOX. We found that CE significantly suppressed mtROS after NLRP3 inflammasome activation (*p* < 0.05; Figures 4E,F).

**Figure 5:**
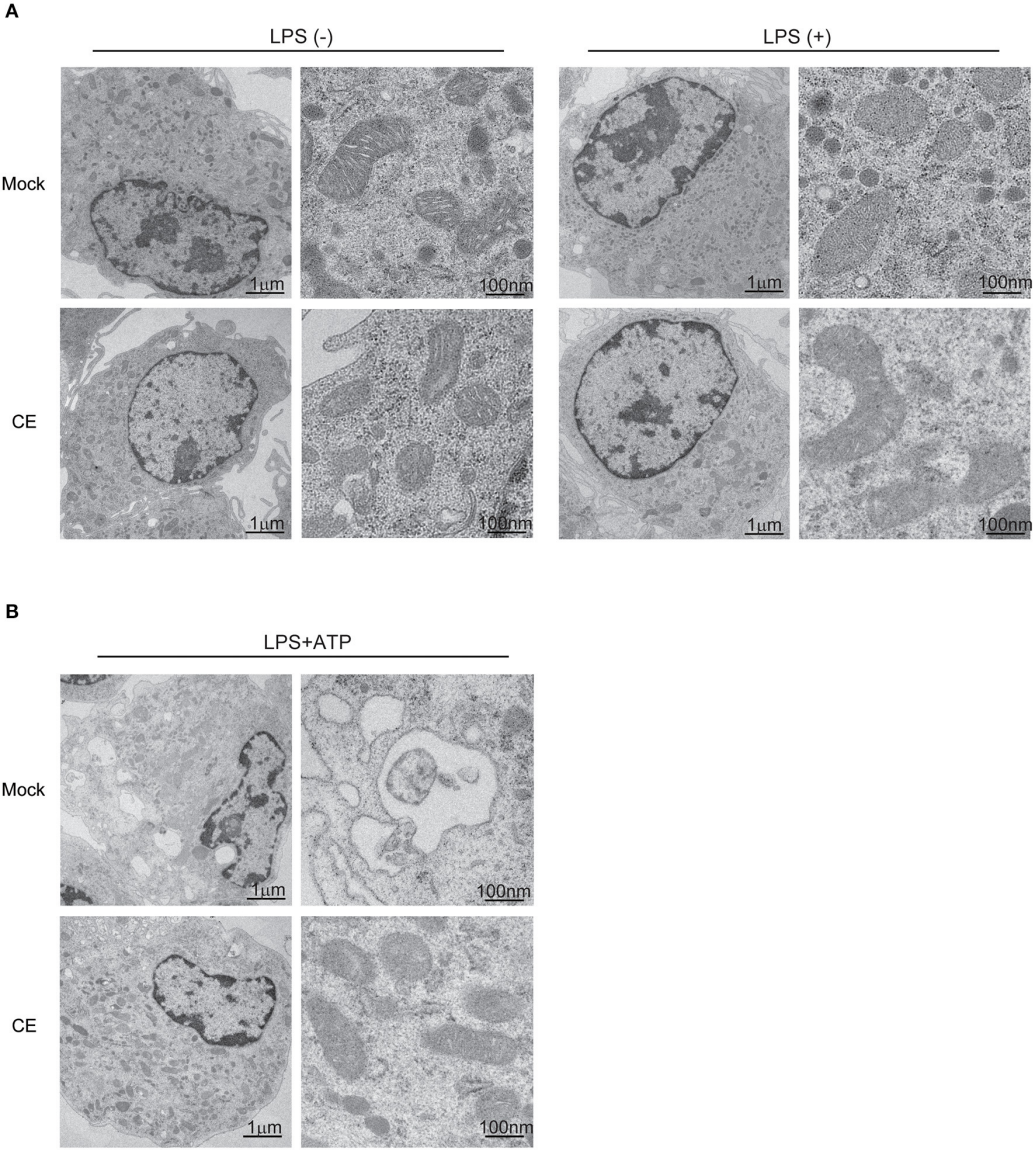
Electron microscope image in murine macrophages. **(A,B)** Electron microscopy images of BMDMs. The images on the right highlight individual mitochondria.

Autophagy is critical for the removal of old or damaged organelles including mitochondria. Several studies have shown that mitochondria damaged by the NLRP3 inflammasome release mtROS and DNA, and are eliminated by autophagy to preserve mitochondrial homeostasis (11, 41). Under unstimulated conditions, we did not observe morphological differences in BMDMs by transmission electron microscopy (Figure 5A). However, we found several autophagic vesicles and a greater abundance of swollen mitochondria in BMDMs without CE after treatment with LPS/ATP (Supplementary Figure S3b). In contrast, CE suppressed NLRP3-induced autophagy and protected mitochondrial morphology (Figure 5B). According to these results, CE selectively suppresses mitochondrial ROS and caspase-1 activation during NLRP3 inflammasome activation.

AIM2 is a cytosolic double-stranded DNA (dsDNA) receptor. It binds to dsDNA and oligomerizes with ASC to initiate the formation of a caspase-1-activating inflammasome, leading to IL-1β secretion (42). Unlike the case for the NLRP3 inflammasome, CE did not affect IL-1β production after AIM2 inflammasome using LPS/poly(dA:dT) stimulation (*p* > 0.05; Supplementary Figures S3a,b). Because CE did not decrease the levels of AIM2, ASC, pro-caspase-1, and cleaved caspase-1 after LPS/poly(dA:dT) stimulation (*p* > 0.05; Supplementary Figure S3c), we thought that CE did not affect AIM2 inflammasome. Taking these findings together, CE selectively inhibits the NLRP3 inflammasome via caspase-1 activation.

### Effect of Carotenoid Supplementation on the NLRP3 Inflammasome

**Figure 6:**
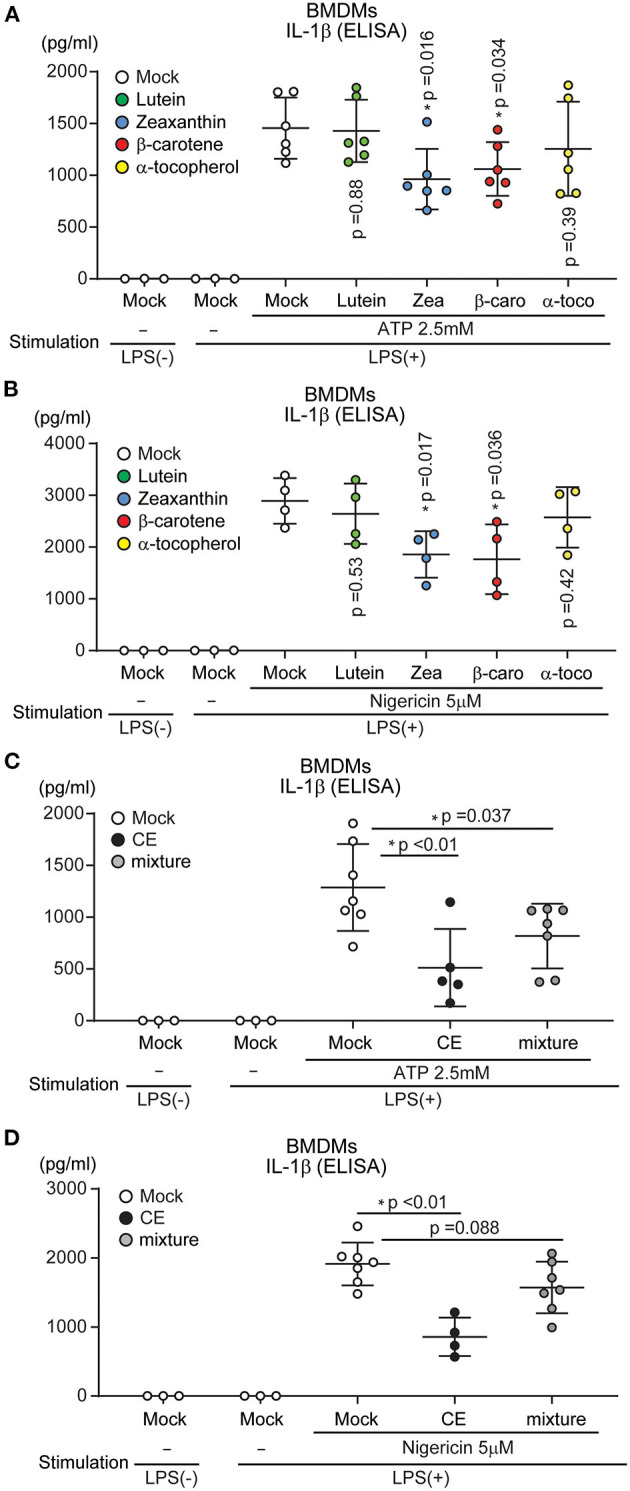
The effect of each component of *Chlorella* extract on IL-1β secretion. **(A–D)** BMDMs were treated with Mock, CE, 1.5 μM Carotenoids (Lutein, zeaxanthin: Zea, β-carotene: β-caro), α-tocopherol (α-toco) and mixture (1.5 μM lutein, 0.12 μM Zea, 0.91 μM β-caro, and 0.16 μM α-toco) for 16 h prior to the addition of 10 ng/mL for 4 h before stimulation with ATP or nigericin for 30 min. The levels of IL-1β in cell culture supernatants were analyzed by ELISA. Error bar show mean ± SEM. *p < 0.05 vs. Mock-treated groups. Data are representative of at least three independent experiments.

The carotenoid-rich extract from *Chlorella* suppressed NLRP3 inflammasome activation. CE suppressed NLRP3 inflammasome activation by targeting mtROS and caspase-1 activation (Figure 4). Although CE contained several carotenoids (Supplementary Table S1), it was unknown which suppressed NLRP3 inflammasome activation. Therefore, we investigated whether individual components (lutein, zeaxanthin, β-carotene, and α-tocopherol) suppressed NLRP3 inflammasome activation. Indeed, supplementation with lutein—the most abundant component of CE—did not significantly suppress LPS/ATP and LPS/nigericin-induced IL-1β secretion (*p* > 0.05; Figures 6A,B). We next examined other components in CE. Although the concentrations were high compared with those in CE, IL-1β production was significantly reduced in BMDMs treated with 1.5 μM zeaxanthin and β-carotene after LPS/ATP and LPS/nigericin treatment (*p* < 0.05; Figures 6A,B). Therefore, we investigated whether a mixture of similar components and concentrations in CE suppressed NLRP3-induced IL-1β secretion. However, a mixture (1.5 μM lutein, 0.12 μM zeaxanthin, 0.91 μM β-carotene, and 0.16 μM α-tocopherol) suppressed LPS/ATP-induced IL-1β secretion (*p* = 0.037), but it was not at the same level as the inhibitory effect of CE (Figures 6C,D). These results indicated that the inhibitory effect of CE on the NLRP3 inflammasome was not an effect of individual components, but the synergistic effect of multiple components in CE.

### *Chlorella* Extract Supplementation Suppresses LPS-Induced IL-1β Production *in vivo*

**Figure 7:**
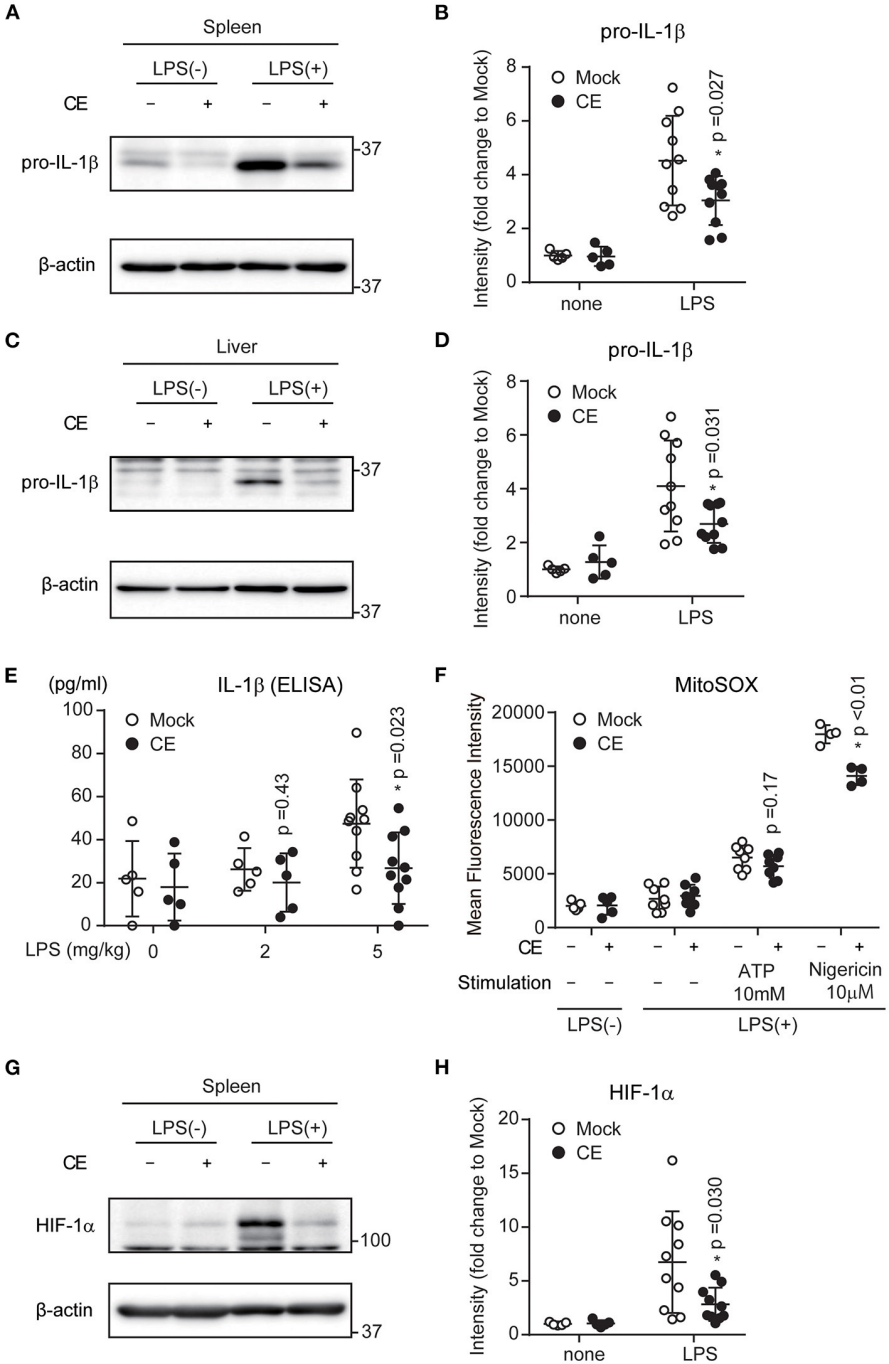
The effect of *Chlorella* extract on LPS-induced IL-1β production *in vivo*. **(A–H)** Mice were injected intraperitoneally (i.p.) with Mock or CE in PBS for 24 h, followed by PBS or LPS (5 mg/kg body weight) for 16 h. Spleens and Livers were isolated, and pro-IL-1β, HIF-1α were analyzed by western blotting **(A,C,G)**. A representative western blotting quantification of pro-IL-1β and HIF-1α **(B,D,H)** was normalized to β-actin expression and relative to Mock only treated group. **(E)** Plasma was isolated from whole blood, and IL-1β production were analyzed by ELISA. **(F)** Mice were injected intraperitoneally (i.p.) with Mock or CE in PBS for 24 h, followed by PBS or LPS (5 mg/kg body weight) for 4 h (*in vivo*), and then collected pMACs. pMACs were stimulated with ATP or nigericin for 30 min, and then were stained with MitoSOX Red for 10 min and then were analyzed by FACS to measure mitochondrial ROS. Error bar show mean ± SEM. *p < 0.05 vs. Mock-treated groups. Data are representative of at least three independent experiments.

Our data indicated that CE suppresses both LPS-induced Il-1b transcription and NLRP3-indeced IL-1β secretion. *In vitro*, macrophages were treated with blood levels of carotenoids when humans ingested chlorella tablets for 1 month. Therefore, to achieve this concentration in mice, CE was administered to mice intraperitoneally. To assess whether CE suppresses IL-1β production *in vivo*, mice were intraperitoneally (i.p.) injected with 300 μg/kg CE 24 h prior to the i.p. administration of 5 mg/kg LPS. CE suppressed LPS-induced pro-IL-1β expression in the spleen and liver (*p* < 0.05; Figures 7A–D). We also found that CE supplementation attenuated IL-1β production in plasma after LPS stimulation (*p* = 0.028; Figure 7E). To assess whether CE suppressed mtROS in mice, the mice were administered intraperitoneally in the following order: 4% thioglycolate solution (3.5 days), mock or 300 μg/kg CE (24 h), and 5 mg/kg LPS (4 h). Four hours after LPS injection, peritoneal cells were collected and cultured for 1 h and then washed five times with PBS. Then, mtROS in adhesive cells (pMACs) were induced by ATP or nigericin. CE also suppressed mtROS in pMACs with nigericin stimulation (*p* < 0.01; Figure 7D). Because CE suppressed LPS-induced HIF-1α induction after LPS stimulation in spleen (*p* = 0.030; Figures 7G,H), CE may also suppress IL-1β production via mtROS- and HIF-1α-dependent pathways *in vivo*.

## Discussion

Here, we demonstrate that CE, which is a food source and nutritional supplement, has selective and potent inhibitory activity against NLRP3 inflammasome activation *in vitro* and *in vivo*. CE may thus be a useful tool for exploring NLRP3 biology and druggability.

In this study, we demonstrated that CE supplementation attenuated LPS/ATP or LPS/nigericin-induced IL-1β secretion *in vitro* (Figure 2). In addition, we showed that treatment with CE reduced plasma IL-1β in an LPS-induced sepsis model (Figure 7). Our study clearly indicated that CE supplementation suppressed mtROS and caspase-1 activation during NLRP3 inflammasome activation (Figure 4). CE inhibited NLRP3 inflammasome activation, but did not affect AIM2 inflammasome activation (Supplementary Figure S3), suggesting that it acts upstream of ASC to suppress inflammasome activation. Because anomalous NLRP3 inflammasome activation is linked to the development of many diseases, several types of NLRP3 inflammasome inhibitor have been developed (2, 43). However, several agents have proven ineffective in clinical settings, and several potential inhibitors require further development (44). None of the small-molecule inhibitors of the NLRP3 inflammasome is currently approved by the US Food and Drug Administration (FDA) (44). Therefore, it is an important finding that CE suppresses NLRP3 inflammasome activation at a range of blood concentrations obtained with s usual dose of Chlorella powder supplement. CE may be more cost-effective than biologic agents and small molecules. In this study, we used a mouse model by a single i.p. injection of sublethal dose of LPS (5 mg/kg). This mice model also recapitulates many cardinal features of neuroinflammation diseases, such as Parkinson and Alzheimer diseases (45, 46). In addition, NLRP3 inflammasome is considered a contributor to the development of neuroinflammation (47, 48). A major question is whether it will be possible to safely and effectively target inflammatory mechanisms that contribute to the pathogenesis of neuroinflammation (49). Because neuroinflammation diseases are chronic degenerative diseases, their treatment requires long-term therapy, imposing a corresponding requirement for a high level of safety. *Chlorella* is generally recognized as safe and few side effects when taken long-term. Therefore, CE may also be an effective supplement of neuroinflammation diseases.

Previous studies showed that LPS-induced HIF-1α promotes IL-1β production by enhancing glycolysis and increasing the level of succinate (6, 50, 51). In this study, we found that CE suppressed Il-1b transcription without suppressing enhanced glycolysis (Supplementary Figure S1c). Because CE did not affect the LPS-induced NF-κB signaling pathway, we expect CE to directly inhibit LPS-induced HIF-1α activation. This CE's suppression of the HIF-1α signaling pathway without affecting the NF-κB signaling pathway will make CE a promising daily supplement that ameliorates or prevents NLRP3-specific diseases without causing immunosuppression. In this study, we could not fully clarify which component of CE suppresses LPS-induced HIF-1α activation. There is thus a need for further research on the mechanism by which CE suppresses LPS-induced HIF-1α activation.

Several studies have shown that lutein supplementation has anti-inflammatory effects that include IL-1β production (52, 53). In this study, in contrast to our expectations, lutein supplementation did not suppress NLRP3-induced IL-1β production. Therefore, we applied a mixture of carotenoids and vitamin E in macrophages. As a result, the mixture of several carotenoids suppressed NLRP3-induced IL-1β production. It has been reported that treatment with lutein at a concentration close to that in our experiment does not have anti-inflammatory effects, but it exerts inhibitory effects on inflammatory mediators when combined with some other components (54). Synergistic effects exerted by a combination of carotenoids and vitamin E have been reported in many studies (55). Thus, the inhibitory effect on NLRP3 inflammasome activation by CE may be a synergistic effect of certain bioactive compounds. Additionally, ~30% of the components in CE were unknown. The mixture of carotenoids significantly inhibited IL-1β, but its inhibitory effect was low compared with CE. Because this may be due to unknown compounds that exert a further synergistic effect, it is necessary to identify these components and investigate their bioactivity.

In this study, we performed intraperitoneal injection of CE rather than oral administration. We conducted all *in vitro* experiments at the concentration of plasma levels of carotenoids after dietary *chlorella* supplementation in humans. In rodents, this blood level concentration of carotenoids may not be achieved *in vivo* without oral administration of at least 1.6 mg carotenoids/day/mouse for ~3 days (56). It was not possible to orally administer such a high level of carotenoids to mice with our prepared CE. Additionally, even when a diet chow with chlorella powder (5%) was fed to mice, the concentration of plasma lutein was approximately one-sixth (data not shown) of that when a chlorella tablet was ingested by humans for 1 month. For these reasons, we chose intraperitoneal administration to mice. However, because chlorella is a health food, it is necessary to examine oral administration in terms of the anti-inflammatory effects by preparing a more concentrated extract from chlorella or dissolving in solutions that are not harmful to animals, such as vegetable oil.

In this paper, we demonstrate that CE suppresses LPS-induced IL-1β transcription and NLRP3-induced IL-1β production. In addition, we show that CE did not affect enhancements of glycolysis and the NF-κB signaling pathway after LPS stimulation. Because CE is more cost-effective than biologic agents, it is a potential therapeutic agent for NLRP3-related diseases with low cost and less immunosuppression. Because we could not show the detailed mechanism by which CE suppresses NLRP3-induced IL-1β secretion, further studies on the relationship between CE and NLRP3 inflammasomes are required.

## Data Availability Statement

The original contributions presented in the study are included in the article/Supplementary Material, further inquiries can be directed to the corresponding author/s.

## Ethics Statement

The animal study was reviewed and approved by the Committee of Ethics on Animal Experiments, Faculty of Medical Sciences, Kyushu University.

## Author Contributions

YN: methodology, validation, formal analysis, investigation, resources, writing—original draft, and visualization. KG: conceptualization, methodology, investigation, resources, writing—original draft, writing—review and editing, visualization, project administration, and funding acquisition. SM, DS, and YT: investigation. TK: resources, supervision, and funding acquisition. DK: conceptualization, writing—review and editing, supervision, project administration, and funding acquisition. All authors contributed to the article and approved the submitted version.

## Funding

This work was supported by JSPS KAKENHI Grant Numbers JP18K11077 and JP16K19196 to KG; and JP20H00530 and JP17H01550 to DK. This work was also supported by a grant from the Takeda Science Foundation, The Shin-Nihon Foundation of Advanced Medical Research and Charitable Trust Laboratory Medicine Research Foundation of Japan (to KG) and research funds from Chlorella Industry Co., Ltd.

## Conflict of Interest

The authors declare that this study received funding from Chlorella Industry Co., Ltd. The funder had the following involvement in the study: materials.

## Publisher's Note

All claims expressed in this article are solely those of the authors and do not necessarily represent those of their affiliated organizations, or those of the publisher, the editors and the reviewers. Any product that may be evaluated in this article, or claim that may be made by its manufacturer, is not guaranteed or endorsed by the publisher.
