# Supplementary material for: Attenuating Effect of Chlorella Extract on NLRP3 Inflammasome Activation by Mitochondrial Reactive Oxygen Species
Source: Front Nutr. 2021 Oct 8;8:763492. doi: 10.3389/fnut.2021.763492 (PMC8531207; doi:10.3389/fnut.2021.763492)
Supplement: Supplementary file 1 [file Data_Sheet_1.docx]

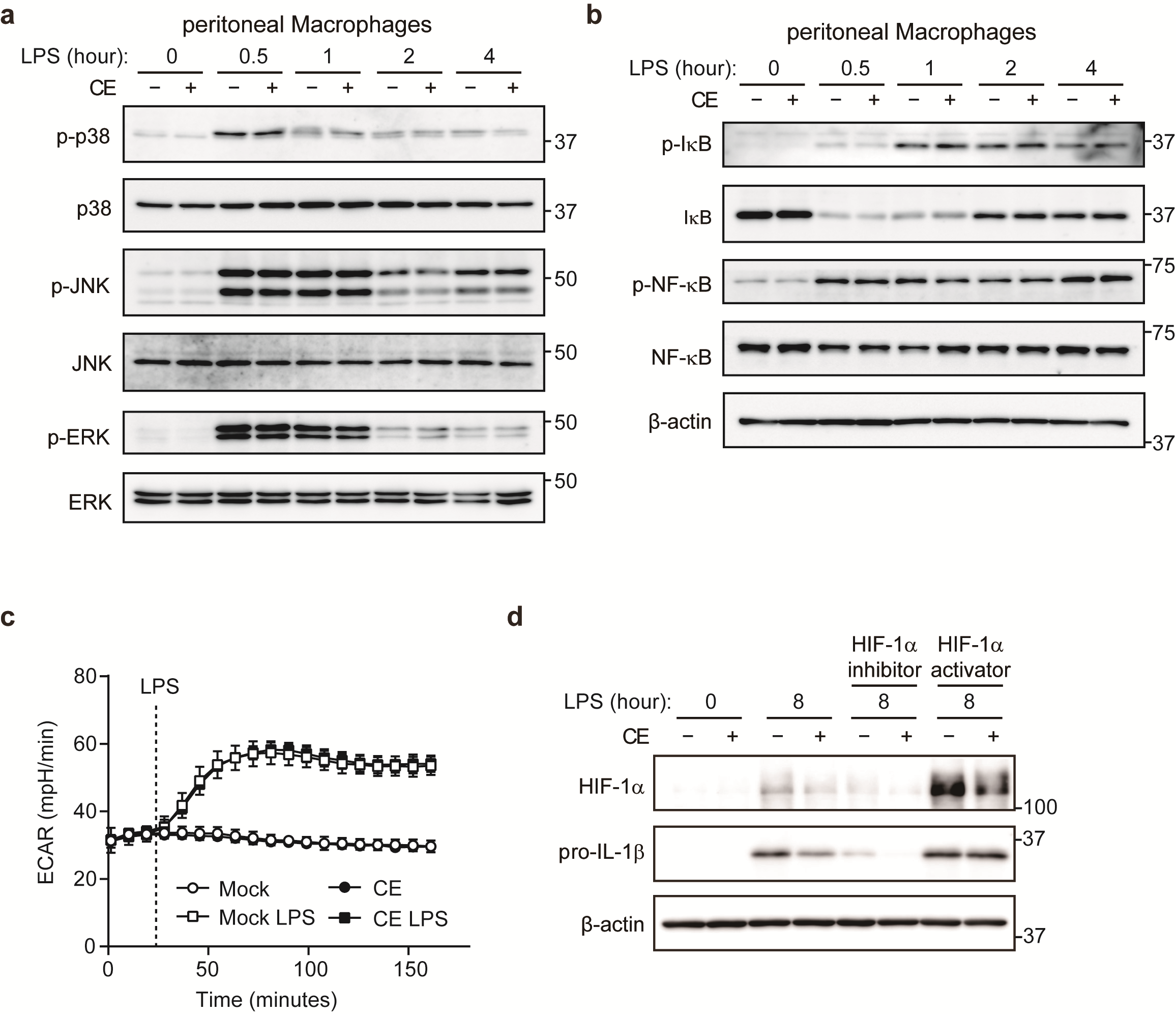


**Supplementary Figure 1
Relationship between HIF-1α and IL-1β in murine macrophages**

(a, b) pMACs were treated with Mock or CE for 16 h before being stimulated with 10 ng/mL LPS for the indicated time periods. Whole cells lysates were analyzed by western blotting for total and phosphorylation of p38, JNK, ERK (a), IκB, NF-κB (b). (c) BMDMs were pretreated with Mock or CE for 16 h before being measured ECAR by seahorse. During seahorse run, BMDMs were injected with PBS or LPS (10 ng/mL). (d) BMDMs were treated with Mock or CE for 16 h before being stimulated with 10 ng/mL LPS for 8 h, at the same time being treated with HIF-1α inhibitor or activator. HIF-1α inhibitor: CAY10585. HIF-1α activator: deferoxamine mesylate. Error bar show mean ± SD. Data are representative of two (c) experiment or three (a, b, d) independent experiments.


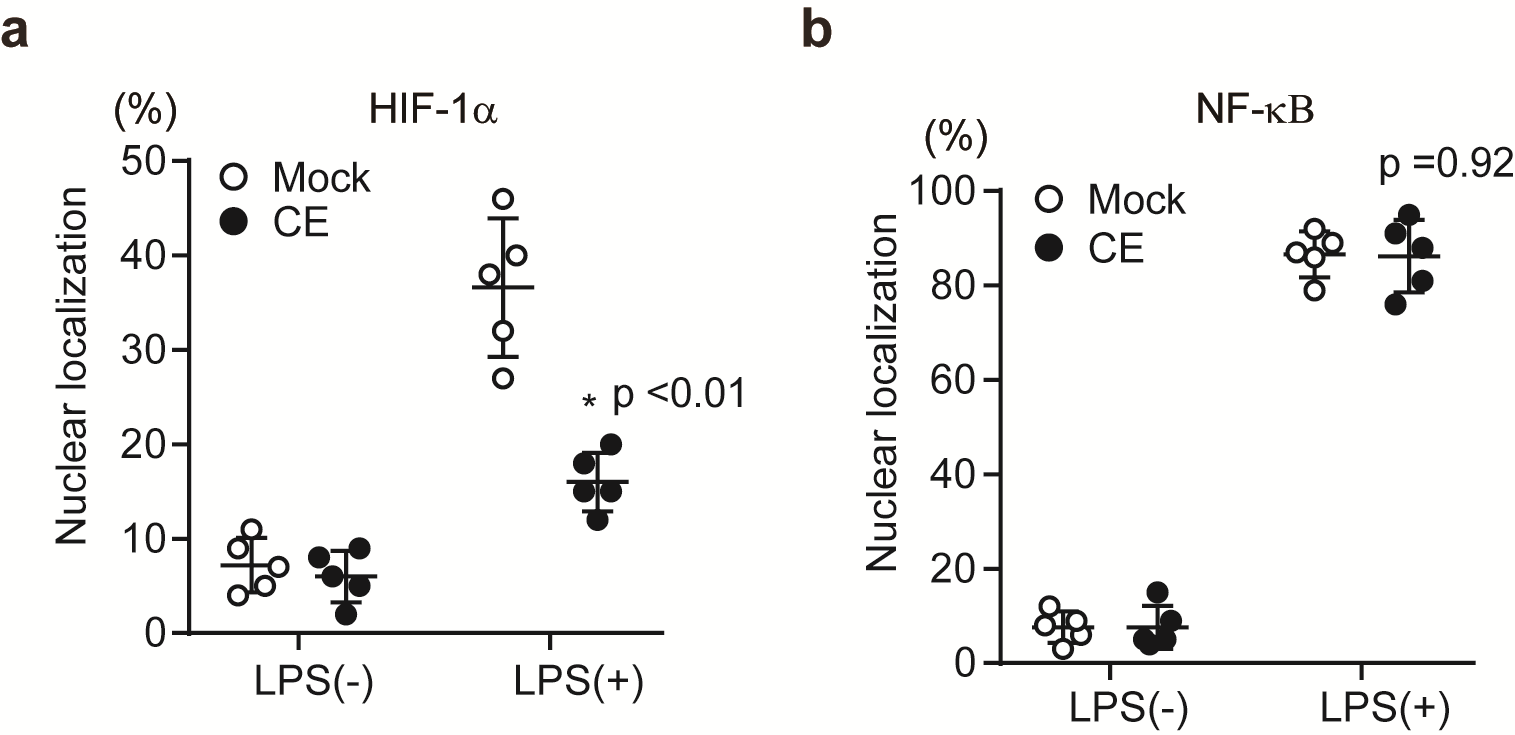


**Supplementary Figure 2
Nuclear translocation of HIF-1α and NF-κB in murine macrophages**

(a, b) BMDMs were treated with Mock or CE for 16 h before being stimulated with 10 ng/mL LPS for 4 h. Subcellular localizations of HIF-1α (a) and NF-κB (b) were performed by immunohistochemistry. The percentage of cells in which HIF-1α or NF-κB merged with DAPI is shown. Error bar show mean ± SEM. *p < 0.05 versus Mock-treated groups. Data are representative of three independent experiments.


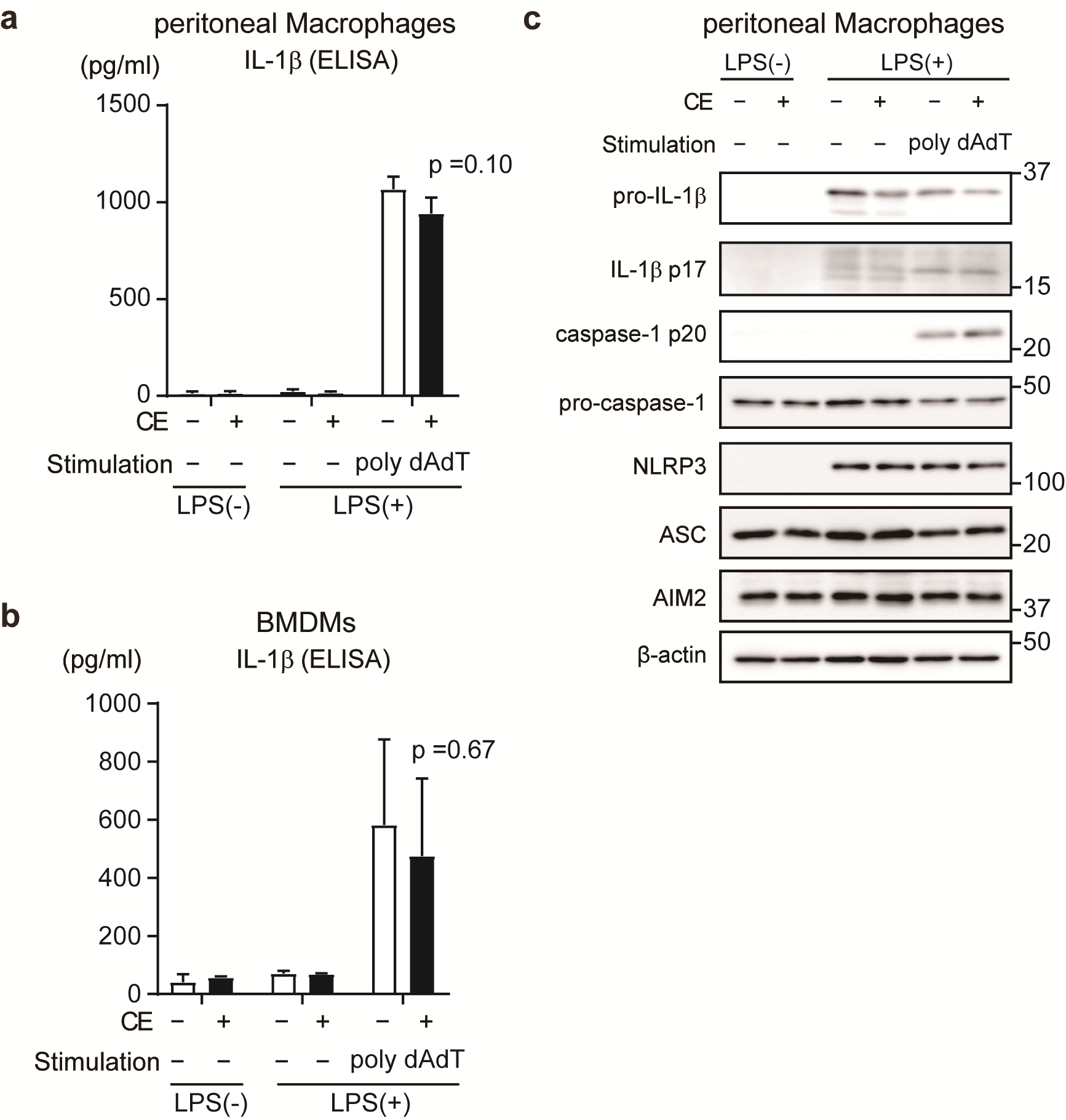


**Supplementary Figure 3**

***Chlorella* extract does not affect AIM2-induced IL-1β production**

(a-c) pMACs and BMDMs were treated with Mock or CE for 16 h prior to the addition of 100 (a, c) or 10 (b) ng/mL for 4 h before stimulation with poly dAdT for 60 min. The levels of IL-1β in cell culture supernatants were analyzed by ELISA (a, b) Whole cells lysates were analyzed by western blotting for pro-IL-1β IL-1β p17, pro-caspase-1, caspase-1 p20, NLRP3, ASC, and AIM2 (c). Error bar show mean ± SEM. Data are representative of two (c) experiment or three (a, b) independent experiments.

**Supplementary Table 1
The composition of Chlorella extract**

| compound | Amount per gram (mg) | concentration of culture medium (μM) |
| --- | --- | --- |
| lutein | 624.3 | 1.500 |
| zeaxanthin | 51.3 | 0.123 |
| α-carotene | 7.7 | 0.019 |
| β-carotene | 35.6 | 0.091 |
| α-tocopherol | 51.3 | 0.163 |
| unknown | 229.9 |  |

**Supplementary Table 2**
**List of chemicals and reagents used in the study**

| REAGENT | SOURCE | IDENTIFIER |
| --- | --- | --- |
| Chlorella powder | Chlorella Industry Co., Ltd. | N/A |
| methanol | Wako | Cat# 137-01823 |
| chloroform | Wako | Cat# 038-02606 |
| ethanol | Wako | Cat# 057-00456 |
| Potassium Hydroxide | Wako | Cat# 168-21815 |
| Sodium Chloride | Wako | Cat# 191-01665 |
| Diethyl Ether | Wako | Cat# 055-01155 |
| Tetrahydrofuran | Wako | Cat# 204-08745 |
| Dimethyl Sulfoxide | Wako | Cat# 049-07213 |
| Lutein | ChromaDex, Inc. | Cat# ASB-00012453-010 |
| Zeaxanthin | Cayman Chemical Co. | Cat# 10009992 |
| β-Carotene | Wako | Cat# 035-05531 |
| α-tocopherol | Wako | Cat# 209-01791 |
| Thioglycollate medium | Nissui Pharmaceutical Co., Ltd. | Cat# 05601 |
| Recombinant Murine M-CSF | Pepro Tech Inc. | Cat# AF-315-02 |
| DMEM | Sigma-Aldrich | Cat# D6046 |
| RPMI1640 | Sigma-Aldrich | Cat# R8758 |
| Penicillin-Streptomycin | Nacalai Tesque | Cat# 26253-84 |
| Adenosine Triphosphate Disodium Salt | Sigma-Aldrich | Cat# A6419 |
| Nigericin sodium salt | Sigma-Aldrich | Cat# N7143 |
| Lipopolysaccharides from Escherichia coli O111:B4 | Sigma-Aldrich | Cat# L2630 |
| MitoSOX^TM^ Red | Thermo Fisher  Scientific | Cat# M36008 |
| Triton X-100 | Sigma-Aldrich | Cat# T8787 |
| Critical Commercial Assays | | |
| REAGENT | SOURCE | IDENTIFIER |
| ELISA MAX™ Deluxe Set Mouse IL-1β | BioLegend | Cat# 432615 |
| RNase-Free DNase Set | QIAGEN | Cat# 79254 |
| PrimeScript™ RT reagent Kit | TAKARA | Cat# RR037A |

**Supplemental Table S3
List of antibodies used for western blot**

| REAGENT | SOURCE | IDENTIFIER | MW(kD) | DILUTION |
| --- | --- | --- | --- | --- |
| anti-IL-1β antibody | R&D systems | Cat# AF-401-NA, RRID:AB_354347 | 17, 31 | 1:2000 |
| anti-Cleaved-Caspase-1 antibody | Cell Signaling Technology | Cat# 89332 | 22 | 1:2000 |
| anti-Caspase-1 antibody | Cell Signaling Technology | Cat# 24232, RRID:AB_2890194 | 48 | 1:2000 |
| anti-NLRP3 antibody | Cell Signaling Technology | Cat# 15101, RRID:AB_2722591 | 110 | 1:2000 |
| anti-ASC antibody | Cell Signaling Technology | Cat# 67824, RRID:AB_2799736 | 22 | 1:2000 |
| anti-AIM2 antibody | Cell Signaling Technology | Cat# 63660, RRID:AB_2890193 | 43 | 1:2000 |
| anti-p-p38 antibody | Cell Signaling Technology | Cat# 4631, RRID:AB_331765 | 43 | 1:2000 |
| anti-p38 antibody | Cell Signaling Technology | Cat# 9212, RRID:AB_ 330713 | 43 | 1:2000 |
| anti-p-JNK2 antibody | Cell Signaling Technology | Cat# 4668, RRID:AB_ 823588 | 46, 54 | 1:2000 |
| anti-JNK2 antibody | Cell Signaling Technology | Cat# 9258, RRID:AB_ 2141027 | 46, 54 | 1:2000 |
| anti-p-ERK antibody | Cell Signaling Technology | Cat# 9106, RRID:AB_ 331768 | 42, 44 | 1:2000 |
| anti-ERK antibody | Cell Signaling Technology | Cat# 9102, RRID:AB_ 330744 | 42, 44 | 1:2000 |
| anti-p-IκBα antibody | Cell Signaling Technology | Cat# 2859, RRID:AB_561111 | 39 | 1:2000 |
| anti-IκBα antibody | Cell Signaling Technology | Cat# 4814, RRID:AB_390781 | 39 | 1:2000 |

| anti-p-NF-κB antibody | Cell Signaling Technology | Cat# 3033, RRID:AB_331284 | 65 | 1:2000 |
| --- | --- | --- | --- | --- |
| anti-NF-κB antibody | Cell Signaling Technology | Cat# 8242, RRID:AB_10859369 | 65 | 1:2000 |
| anti-HIF1 antibody | abcam | Cat# ab179483, RRID:AB_ 2732807 | 110 | 1:2000 |
| anti-β-actin antibody | Merck (Sigma-Aldrich) | Cat# A5441, RRID:AB_476744 | 42 | 1:5000 |

**Supplemental Table S4
List of primer sequences used for RT-PCR analysis**

| Primer name | Sequence 5’→3’ |
| --- | --- |
| 18S Forward | CGCGGTTCTATTTTGTTGGT |
| 18S Reverse | AGTCGGCATCGTTTATGGTC |
| Il-1b Forward | GCTTCAGGCAGGCAGTATCAC |
| Il-1b Reverse | CGACAGCACGAGGCTTTTT |
| Il-6 Forward | TGATGCACTTGCAGAAAACA |
| Il-6 Reverse | ACCAGAGGAAATTTTCAATAGGC |
| Tnfa Forward | ACAAGGCTCTGCCCCGACTAC |
| Tnfa Reverse | TGGAAGACTCCTCCCAGGTATATG |
| Ifn-b1 Forward | CTGGCTTCCATCATGAACAA |
| Ifn-b1 Reverse | CATTTCCGAATGTTCGTCCT |
| Nos2 Forward | CGAAACGCTTCACTTCCAA |
| Nos2 Reverse | TGAGCCTATATTGCTGTGGCT |
